# Response to Letter

**DOI:** 10.1111/mcn.13045

**Published:** 2020-07-03

**Authors:** Sarah L. Dalglish, Naoko Kozuki

**Affiliations:** ^1^ Johns Hopkins Bloomberg School of Public Health Johns Hopkins University Baltimore Maryland USA; ^2^ Research and Innovation Department International Rescue Committee Washington DC USA

We appreciate the writers' taking time to engage with our research, as well as the important work their organization does around the globe. The World Food Programme's (WFP) nutrition programmes are critical to the survival of millions of vulnerable children.

In our study, we triangulated data collected through interviews with programmatic and policy documents, as well as between respondents themselves, to ensure the accuracy of the information we reported. Regarding the disputes of fact conveyed by the authors of the letter, we investigated the claims in our original data and by reaching out to informants currently working in these settings.

On the WFP budget in Nigeria, regional budget shortfalls were mentioned by multiple stakeholders interviewed in our study, as well as in published news reports from that time. We appreciate the point that sufficient donor funds were available to cover planned caseloads; we meant to highlight that most children with moderate acute malnutrition (MAM) outside of north‐east Nigeria did not receive any treatment, though of course this is not the sole responsibility of WFP. As noted by the writers, this situation is due in part to the Nigerian Community Management of Acute Malnutrition (CMAM) protocol's focus on children with severe acute malnutrition (SAM) children, which was under review at the time of writing. We concede that this nuance could have been made clearer in our article.

Regarding restrictions to subcontract monitoring of WFP in South Sudan, we confirmed with informants currently in the field that indeed, WFP South Sudan does not have restrictions to subcontract monitoring. A draft report containing this assertion was reviewed for accuracy by multiple WFP stakeholders, both at headquarters and in the South Sudan office, but this discrepancy was not highlighted. Nonetheless we regret the error.

Finally, the writers state that RUSF was not the only food supplement available for treatment of children with MAM. The focus of this study remained on ready‐to‐use therapeutic food (RUTF) and ready‐to‐use supplementary food (RUSF) available in the context of the Combined Protocol; therefore, we did not focus on Blanket Supplementary Feeding Program (BSFP) or other programmes that lie outside that purview. The content of our article does not refute availability of Super Cereal Plus or BSFP programmes for MAM treatment.

WFP is recognized globally for its work to feed and care for malnourished children. We look forward to continued discussions on how to prevent and treat the scourge of malnutrition, particularly in conflict‐affected contexts, and to ongoing constructive collaboration with all partners.

